# The influence of mood and social relationships on the intensity of basic self-disturbance: an experience sampling method investigation

**DOI:** 10.3389/fpsyt.2025.1514351

**Published:** 2025-04-08

**Authors:** Lise Baklund, Jan Ivar Røssberg, Sigurd Arne Melbye, Henri Pesonen, Paul Møller

**Affiliations:** ^1^ Department of Mental Health Research and Development, Division of Mental Health and Addiction, Vestre Viken, Drammen, Norway; ^2^ Oslo University Hospital Division of Mental Health and Addiction University of Oslo, Institute of Clinical Medicine, Oslo, Norway; ^3^ Oslo Centre for Biostatistics and Epidemiology, Oslo University Hospital, Oslo, Norway

**Keywords:** basic self-disturbance, adolescents, clinical high risk (CHR) for psychosis, mood, psychosocial factors

## Abstract

**Background:**

Basic self-disturbance (BSD), the overarching concept of various experiences of self-alienation, referred to as anomalous self-experiences (ASEs), is considered a relatively stable clinical marker for the potential development of schizophrenia spectrum disorders. However, research on BSD in children and adolescents in the pre-psychotic phase is limited. Research on individuals at risk for psychosis shows that psychosocial factors are critical in psychosis development, but studies of these factors and the relationship between psychosocial factors and the severity of ASEs are lacking. The present study aims to investigate the extent to which mood, social relationships, and psychosocial factors contribute to the development of BSD in adolescents at risk for psychosis.

**Methods:**

We used the experience sampling method to collect real-time data from 27 help-seeking 12- to 19-year-old adolescents. We analyzed data from daily registrations on a smartphone app, measuring the intensity of BSD, mood, and social settings over various time periods (hourly, daily, and weekly over 6 months) by linear mixed regression modeling.

**Results:**

Negative emotions were positively associated with the mean ASE scores (β = 0.30, 95% CI = (0.26, 0.34), whereas for the positive emotions, the contrast of the association was significantly negative [β = −0.57, 95% CI = (−0.63, −0.51)]. The effect of being alone at the time of the response on the intensity of ASEs compared to being with others was significantly positive [β = 0.27, 95% CI = (0.08, − 0.46)]. However, this effect was observed only when not being at home, as indicated by the effect of being at home [β = −0.04, 95% CI = (−0.09, 0.11)] compared to not being at home and the interaction between the two social context variables [β = −0.20, 95% CI = (−0.44, −0.04)].

**Conclusions:**

Mood and social settings appear to be influencing factors in the expression and intensity of ASEs. These factors should be addressed in the clinical approach to BSD, and further studies investigating the influence of various psychosocial factors on BSD experiences should be conducted.

## Introduction

Since the mid-1990s, research on early identification and the development of targeted interventions in the pre-psychotic phase has increased. The so-called Clinical High-Risk (CHR) criteria are the most widely accepted criteria to determine significant clinical risk for psychosis. However, based on prediction studies (1–4), there are several challenges concerning the specificity and sensitivity of these criteria. The majority of the individuals fulfilling the criteria do not develop psychosis, but there is a lack of evidence for any specific type of psychotic disorder predicted by the CHR criteria (5), and, finally, the CHR criteria have been found to be predictive of psychosis in clinical samples only (6).

### Basic self-disturbance in adolescents at risk for psychosis

Several studies have pointed out that basic self-disturbance (BSD), also called anomalous self-experience (ASE), or self-disorder (SD), a core phenotypic marker of schizophrenia, also constitutes an early, pre-psychotic risk marker (7). BSD refers to a profound alteration of subjectivity in terms of the pre-reflective sense of selfhood and the crucial sense of existence as a vital and self-identical (“I am myself”) subject of experience (8, 9). The normal experience of oneself as a coherent and temporally continuous unity is partly and gradually replaced by confusing experiences of oneself as fragmented with diminished presence and discontinuity in time, space, and agency. Intriguing experiences of self-transformation, depersonalization, and derealization are typically expressed by patients through statements like “I feel like I’m not a human. I don’t know what I am” and “Nothing seems real, not even my thoughts, in fact almost the whole world” (10). While BSD is the overarching concept for these characteristic experiences, ASEs refer to the various forms of BSD as defined by the phenomenological checklist Examination of Anomalous Self-Experience (EASE) (11). In studies of CHR individuals, high levels of ASEs have been found to predict non-remission of CHR status, psychosis transition (12, 13), and predominantly schizophrenia spectrum disorder (SSD), and to be strongly associated with negative symptoms (14), global dysfunction (15), and suicidality (16), as measured by standard clinical structured interviews.

Prospective studies have investigated BSD from baseline to follow-up periods ranging from 1.5 to 7.5 years. Many studies have demonstrated a certain temporal stability in both patients with first-episode schizophrenia and individuals at CHR, indicating that BSD is a trait-like phenomenon (13, 17, 18). BSD has been shown to aggregate in CHR individuals as compared to controls (19) and to predict SSD in adulthood (13). Therefore, the question of whether BSD may be a closing-in strategy for subgroups of CHR has been raised (12, 15). However, recent studies have reported various trajectories, which include limited mean decrease and even “remission” of ASEs from baseline to follow-up in patients with schizophrenia (20) and in CHR-P patients (21, 22). Nevertheless, the mechanisms that may bring some individuals from ASEs to psychosis and SSD, while in others ASEs taper off to insignificant levels or remission, remain unknown.

Few studies have been conducted on BSD in childhood and adolescence, and there is a lack of substantial evidence on BSD as a trait phenomenon in individuals under the age of 18. The fact that children and adolescents are constantly developing represents a unique aspect that must be accounted for in research and treatment. In the last few years, in addition to research on traditional psychopathological symptoms, psychosocial aspects have been given more attention. Studies have found more psychosocial stress, interpersonal sensitivity, negative emotions, and social withdrawal in CHR individuals compared to controls (23, 24). Significant life events (i.e., change of school, parental divorce, and somatic illness) and social relationships and situations (i.e., lack of close friends or supportive family environment) are examples of factors that appear to have a negative impact on psychotic symptoms. Further, brief psychotic symptoms in CHR individuals are associated with higher levels of negative affect than psychotic symptoms in individuals with stable psychosis, indicating that subclinical psychotic experiences may be stressors in themselves (25). Still, clear descriptions of the underlying interrelations between psychopathology, emotions, and social stress in early pre-psychotic phases are lacking. Thus, for early intervention in adolescents, we need to know more about the course of BSD and the relationship between BSD and environmental and contextual factors. Considering the normal development of rapid shifts in mood, behavior, and psychosocial functioning during adolescence, we should look closely at contextual influences from both short- and long-term perspectives. The aim of the study was to investigate the relationship between ASEs, on the one hand, and different mood conditions and social environments/relations in everyday life, on the other hand, in adolescents at CHR.

We posed the following research questions (RQs):

During a period of 6 months:

RQ1: Are negative and positive emotions associated with the intensity of ASEs?

RQ2: Is being alone associated with a higher intensity of ASEs than being with others?

RQ3: Is being at home associated with a higher intensity of ASEs than being away from home?

## Materials and methods

### Design

This is a prospective naturalistic multi-case study investigating individual courses of ASEs during 6 months in 12–19-year-old help-seeking adolescents considered to be at risk for psychosis. Detailed knowledge about the prospective course of ASEs in adolescents requires methods that can capture the dynamic processes of subjective experiences and environmental factors in everyday life, and the experience sampling method (ESM) is tailored for this purpose. It is a mobile assessment approach, or a diary method, designed to measure thoughts, mood, symptoms, and contextual information in the real-time flow of daily life. We used data from daily registrations of ASEs, simultaneously with mood (negative and positive), social relationships (alone and together with others), and type of situation/location (at home and away from home) on a smartphone app.

### Patient recruitment and settings

Patients were recruited from specialized outpatient units in seven Child and Adolescent Mental Health Services (CAMHS) and one Adult Mental Health Service from three different Hospital Trusts in South-Eastern Norway. Mental health care in Norway is organized in geographical catchment areas, where all inhabitants are offered public health care, which diminishes socio-economic bias. Information about the research project was distributed to clinicians in the clinical units through informational meetings and by brochures and mail correspondence where clinicians were encouraged to ask patients aged 12–19 with clinical suspicion of psychosis to participate in the study.

### Inclusion and exclusion

We applied an extended version of the Prodromal Questionnaire 16-item version (PQ-16) (26), supplemented with four ASE questions, adapted from the EASE manual by one of its main authors (PM), and based on frequently reported EASE items in six studies (8, 12, 18, 19, 27, 28). Exclusion criteria were established psychosis, intellectual impairment, neurological or developmental disorders, current antipsychotic treatment (current or for ≥4 weeks of life, equivalent to a dose of ≥5 mg olanzapine per day), clearly substance-induced CHR symptoms, or not being fluent in the Norwegian language. Intellectual impairment, neurological and developmental conditions, and any substance use were assessed by the clinicians who recruited the patients prior to the research interviews. The patients participated in the study on the condition of informed consent (for patients under 16 years of age, both parents also consented). Clinically referred patients who also endorsed six or more items on the PQ-16 part of the prodromal screening instrument and additionally asserted at least one clearly positive answer to the four ASE questions in the screening instrument continued to the Structured Interview for Psychosis-risk Syndrome (SIPS). If patients confirmed one of the CHR syndromes on the SIPS, the patients continued to a full EASE interview. Next, if the patients clearly confirmed at least three prototypical ASEs on the EASE, the patients were included in the study. The transcripts/summaries of the full EASE interviews were read, discussed, and re-confirmed by the patients themselves, after which the patients chose three personally significant and well-manifested ASEs, formulated as statements/verbatim citations to register over the phone in accordance with the ESM schedule.

The study was approved by the Regional Committee of Medical and Health Research Ethics (Id. no. 2016/1758c). The regional agency of the Norwegian Data Protection Authority (NSD) was notified.

### Measures

#### Diagnoses

As the majority of mental disorders in childhood and adolescence have high levels of comorbidity and limited temporal stability, we chose to report the principal clinical diagnoses (more than one, if applicable) achieved through all-data consensus assessment based on the ICD-10 in multidisciplinary clinical teams at the sites.

#### The Structured Interview for Psychosis-risk Syndrome

Patients fulfilling the Criteria for Psychosis-risk Syndromes (COPS) in the SIPS (29) were included. LB conducted the interviews. The inter-rater reliability (IRR) on the SIPS was tested by comparing the scores on nine case vignettes with the raters’ final scores from the North American Prodrome Longitudinal Study (NAPLS). CHR status agreement was 100%, and the Scale of Prodromal Symptoms (SOPS) positive symptom score IRR was excellent [single measure intraclass correlation (ICC): 0.95, 95% CI: (0.96, 0.99), two-way mixed-effects model, absolute agreement].

#### Examination of Anomalous Self-Experience

The EASE interview consists of 57 items divided into five rational domains: 1) cognition and stream of consciousness, 2) self-awareness and presence, 3) bodily experiences, 4) demarcation/transitivism, and 5) existential reorientation. The EASE has very good internal consistency and inter-rater reliability (30). The patients were asked to describe their experiences in detail for each item in their own words and to give concrete examples. All EASE interviews were conducted by LB and videotaped; after scoring, they were all evaluated, discussed, and confirmed/disconfirmed in close dialog with PM (one of the main authors of the EASE) as part of continuous supervision.

### Sample size and the ESM schedule

The time intervals for typical ESM studies are often short (7–10 days) with frequent measures each day (6–10). In order to avoid bias from measuring ASEs in a period that would not be representative of the daily life of the participants (vacation, other illness, time off from school, etc.), we decided to measure ASEs during two different periods: the first during seven consecutive days and the second during 6 months. We planned to 1) prompt each participant 10 times a day in the 1-week schedule and 2) 10 times on a single day every second week in the 6-month (24-week) schedule, providing a total of 70 and 120 measurement points per participant, respectively. Recommended sample sizes in ESM research rely on previous simulation research aimed at developing rule-of-thumb criteria for sample size considerations in multilevel designs. According to the recommendations of Kreft (31) and Oleson et al. (32), a number of 30 at the macro level (our participant level) and a number of 30 at the micro level (our within-subject repeated-measures level) are sufficient in ESM studies. This leads to a desired total sample size of 900, which in a two-level approach is defined as the product sum of N × n (30 × 30). Since our study was designed to investigate ASEs over a longer time period (24 weeks), we considered the sample to be sufficient based on the recommendations in the “ESM rules”. First, in a 1-week ESM schedule, the app was set up to emit a signal at randomly selected time points between 7:30 AM and 10:30 PM (15 h) within mean intervals of 90 min(range, 15 min to 3 h) 10 times a day for seven consecutive days. Second, in the 6-month schedule, the app was set up to emit a signal at randomly selected time points within the same one-day interval frame, but this time only during one (randomly allocated) single day every other week for 6 months. With 30 participants in the study, the total multilevel sample sizes (the total amount of measurements) for the two schedules were 2,100 and 3,600, respectively, which we considered to be well above that recommended by Kreft’s 30/30 rule, and which also allowed for some degree of missing data. We included 30 adolescents at baseline. Of the 30 participants, three withdrew before starting the app registrations, and the “final” sample included 27 participants.

#### The app questionnaire

We developed an ESM questionnaire that was installed, through an encrypted and secure solution, on a smartphone app specifically developed for our study and provided by Services for Sensitive Data (TSD) at the University of Oslo (UiO). The app submitted the data to a form in Nettskjema running on https://nettskjema.uio.no. At each prompt, the participants were instructed to respond to (fill in) the ESM questionnaire immediately. The ESM questionnaire asked for 1) levels of intensity and distress for each of the three personal ASEs, marked on a vertical scroll bar on a 7-point Likert scale (0 = not at all and 6 = very much); 2) nine mood statements (happy, relaxed, content, insecure, lonely, anxious, irritated, sad, and guilty) (7-point Likert scale); 3) closed-ended questions about the type of situation/setting the participant was in at the time of the response (at home, at school, visiting others, together with a partner, and in public places); and 4) what kinds of social relationships (alone, with friends, with classmates, with family, and with a partner). It took approximately 2 minutes to fill in the questionnaire. If the participants did not fill in the questionnaire within 15 minutes, it was closed until the next prompt. To ensure that the participants mastered the app and the ESM questionnaire, LB contacted each participant on the second day of the 1-week registration period and after 1 month in the 6-month period. After 6 months of ASE registration on the app, the patients were invited to finalize the follow-up with SIPS and EASE interviews.

### Statistical analyses

The observations used in the statistical analyses were the mean intensities of the three individually selected ASEs by each participant, mood questionnaire outcomes in the categories of positive and negative emotions, and the social context indicator variables in the categories of whether a respondent was alone/not alone and at home/not at home. In the analyses, ASEs were the dependent variable, whereas mood and social context were the independent variables. Measurements were repeated observations from the study period. The intensities of ASEs, which were on a 7-point Likert scale, were handled as numerical variables and averaged (across the three ASEs) into a single observation per time point. Similarly, the 7-point Likert scale mood questionnaire outcomes were averaged into positive and negative mood groups per time point. The “positive” moods were happy, relaxed, and content, and the “negative” moods were insecure, lonely, anxious, irritated, sad, and guilty. Social context indicator variables were factors per time point.

Graphical explorative analyses were used to investigate population- and individual-level associations of the variables. Scatterplots of mood scores vs. ASE scores with population- and individual-level linear regression lines indicate general associations in the data. Social context-wise interquartile range (IQR) interval plots were used to illustrate the individual-level variability in mean ASEs along with the effect of social contexts per patient. In addition to explorative analysis, linear mixed regression modeling was used due to the repeated-measures design of the study. The average ASE was the outcome, but four different models, defined by the selection of explanatory variables, were fitted. Each of the models contains a patient-specific random intercept. In Model 1, the explanatory variables were the mood scores, an indicator variable for positive and negative mood, and the interaction of the average score and the positive/negative indicator variable. In Models 2 and 3, at home/not at home and alone/not alone—indicator variables—were used as the sole explanatory variables, respectively. Finally, in Model 4, both social context variables and their interaction were included as explanatory variables. All of the statistical analyses were carried out using R 4.4.0 using the tidyverse and lme4 libraries (33–36).

## Results

**Figure 1:**
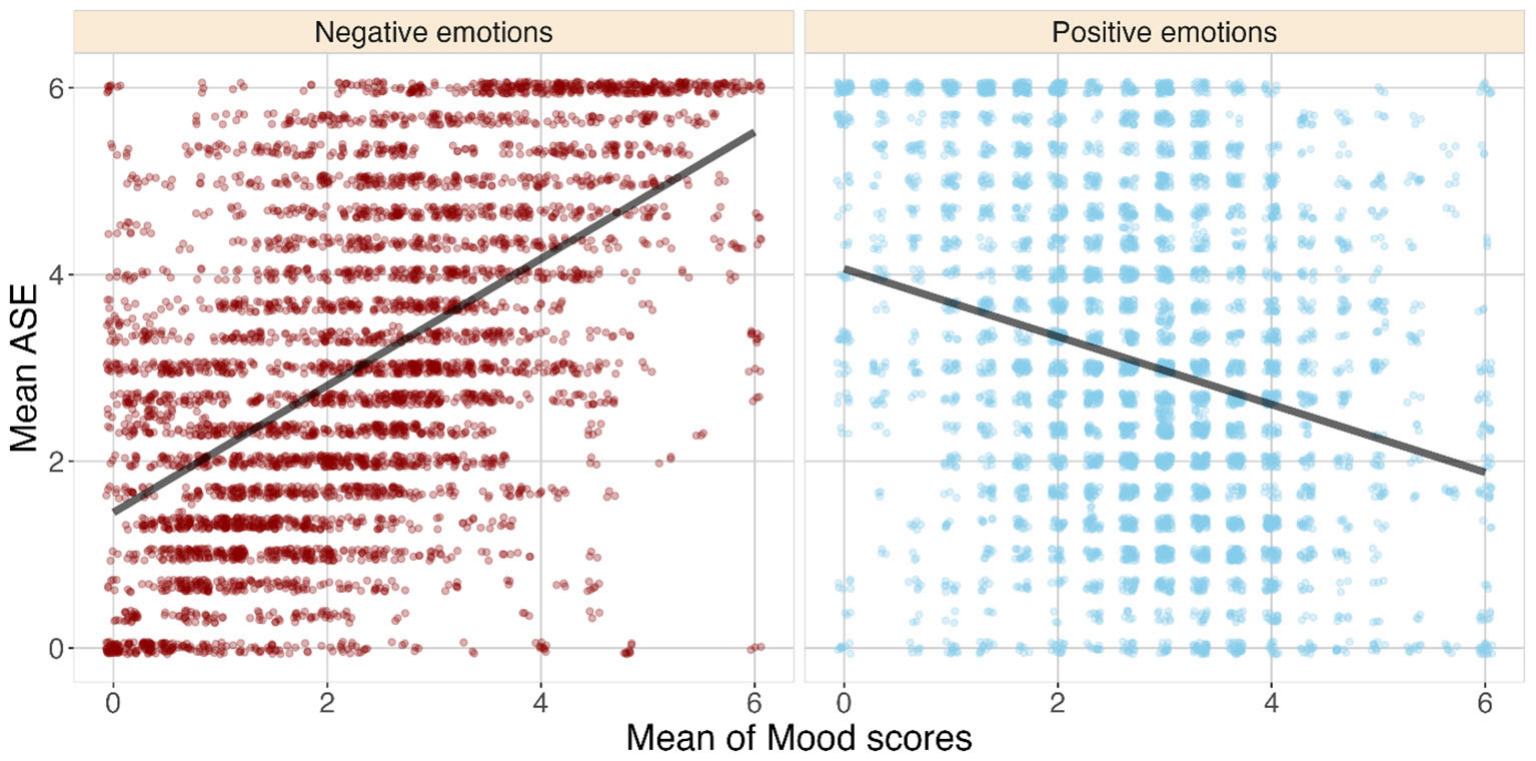
Scatterplot of the mean of the mood scores partitioned into negative and positive moods versus the mean of the anomalous self-experience (ASE) scores. Narrower lines are linear regression lines for each of the patients, and the thick line is the population-level linear regression line.

Sociodemographic and clinical characteristics are provided in **Table 1**. The average number of responses for the full 6-month period was 64.93 [95% interpercentile interval: (25.95, 127)], and the average length of participation in the study was 176.41 days [95% interpercentile interval: (62.85, 300.65)]. The time intervals were defined as the number of days between the first response and the last response. In **Figure 1**, the means of the mood scores for positive and negative emotions are plotted against the means of the ASEs at the time of the response (RQ1). The individual linear regression lines for each patient illustrate the subject-level effect. Our results show that the average mood scores of the negative emotions are associated with larger means of ASE scores, and for positive emotions, the association was in the opposite direction.

**Table 1:** Sociodemographic and clinical characteristics.

All participants, n = 27		
Female subjects, N (%)	16 (59.3)	
Age, mean (SD)	16.1 (1.2)	
School attendance		
Full-time, N (%)	22 (81.4)	
Part-time, N (%)	2 (7.4)	
Drop-out, N (%)	3 (11.1)	
ICD-10 diagnosis	Baseline* (N = 27)	Follow-up* (N = 23)
F20–29 Schizophrenia, schizotypal and delusional disorders, N (%)	0 (0)	2 (8.7)
F30–39 Mood [affective] disorders, N (%)	5 (18.5)	4 (17.4)
F40–48 Neurotic, stress-related, and somatoform disorders, N (%)	16 (59.3)	9 (39.1)
F60–69 Adult personality and behavior disorders, N (%)	0 (0)	1 (4.3)
F90–99 Behavioral and emotional disorders with onset usually occurring in childhood and adolescence, N (%)	4 (14.8)	4 (17.4)
Missing, N (%)	2 (7.4)	3 (13.0)
	Baseline (n = 27)	Follow-up (n = 23)
SIPS, mean (SD)	33.9 (8.5)	28.6 (12.2)
EASE continuous (range 0–4), mean (SD)	46.9 (15.7)	32.5 (20.2)
EASE dichotomized (range 0–1), mean (SD)	16.4 (6.1)	11.3 (7.8)

SIPS, Structured Interview for Psychosis-risk Syndrome; EASE, Examination of Anomalous Self-Experience.

*Number of patients.

**Figure 2:**
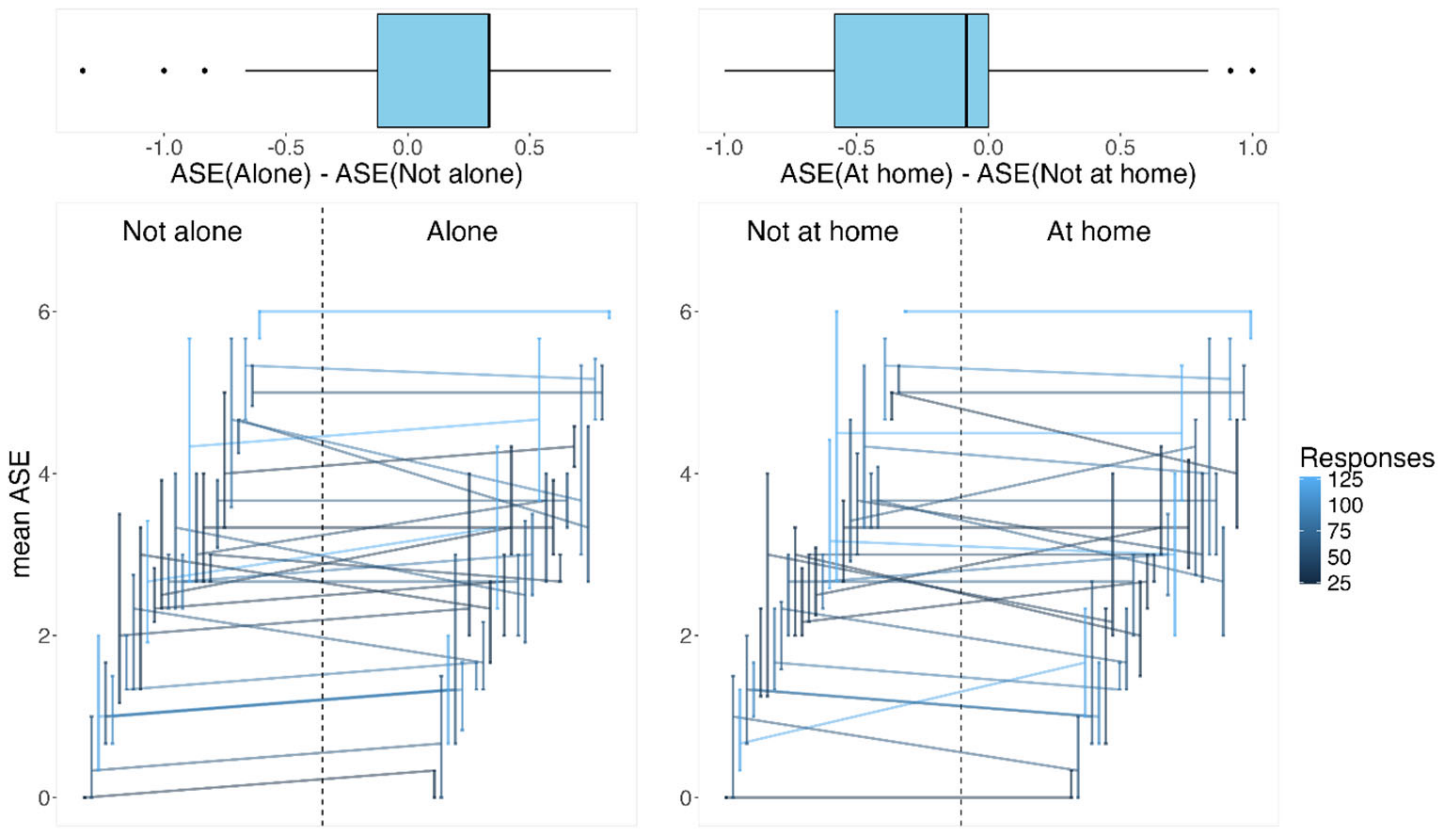
On the bottom: the horizontally shifted vertical lines represent interquartile ranges in two social contexts of the mean anomalous self-experience (ASE; for each patient). The patient-specific median ASEs in these contexts are connected by lines to connect the patient-specific data and to indicate the difference in the contexts: not alone versus alone and not at home versus at home. On the top: the differences in the medians for each patient in the two social contexts are illustrated via boxplots.

In **Figure 2**, the distributions of the mean ASE scores are illustrated patient-wise using medians and IQRs in two different social contexts defined by being at home versus not being at home and being alone versus not being alone. Visually, the data indicate that on average, patients’ average ASE scores increase when being alone compared to not being alone and decrease when not at home compared to being at home.

The results of the four linear mixed regression models are reported in **Table 2** along with the marginal and conditional R^2^ coefficients, which indicate that population-level effects explain very little of the mean ASE variability. Instead, it is the patient-specific random effects that explain the majority of the variability. Model 1 indicates that the average mood scores have a statistically significant association with the mean AES score. The estimates of the joint effects Mean of mood score × Mood indicate that the scores of each of the positive emotions have a negative association with the mean ASE score. Negative mood is chosen as the baseline in the model [β = 0.30, 95% CI = (0.26, 0.34)]. The contrast Mean of mood scores × Mood (Positive) is negative compared to negative mood score effects [β = −0.57, 95% CI = (−0.63, −0.51)]. This indicates that the positive moods have a statistically significant negative association with the mean ASE scores. Model 2 indicates that there is no statistically significant effect of being at home vs. not being at home on the mean ASE score by β = −0.06, 95% CI = (−0.16, 0.05) at the population level (the full sample). Model 3, in contrast, indicates that the effect of being alone vs. not being alone is a little higher: β = 0.09, 95% CI = (−0.01, 0.20). Model 4 includes both social contexts and their interaction in the same model. Being alone has a significantly increasing effect on the mean AES score by β = 0.27, 95% CI = (0.08, 0.46), but the effect vanishes almost completely when being alone at home [being at home: β = −0.04, 95% CI = (−0.19, 0.11); the interaction effect β = −0.20, 95% CI = (−0.44, 0.04)].

**Table 2:** Estimated fixed-effects coefficients from linear mixed regression models.

	β	95% CI	p-Value
Model 1			
(Intercept)	2.19	(1.72, 2.67)	**<0.001**
Mean of mood scores	0.30	(0.26, 0.34)	**<0.001**
Mood (positive)	1.46	(0.13, 1.62)	**<0.001**
Mean of mood scores × Mood (positive)	−0.57	(−0.63, −0.51)	**<0.001**
**Marginal R^2^/conditional R^2^: 0.056/0.625**			
Model 2			
(Intercept)	2.94	(2.45, 3.44)	**<0.001**
At home	−0.06	(−0.16, 0.05)	**0.297**
Marginal R^2^/conditional R^2^*: 0.001/0.609			
Model 3			
(Intercept)	2.86	(2.36, 3.37)	**<0.001**
Alone	0.09	(−0.01, 0.20)	**0.079**
**Marginal R^2^/conditional R^2^: 0.000/0.607**			
Model 4			
(Intercept)	2.88	(2.38, 3.39)	**< 0.001**
Alone	0.27	(0.08, 0.46)	**0.006**
At home	−0.04	(−0.19, 0.11)	**0.603**
Alone × At home	−0.20	(−0.44, 0.04)	**0.108**
**Marginal R^2^/conditional R^2^: 0.002/0.609**			

The response variable in all models is the mean of three anomalous self-experience (ASE) scores. Model 1: The baseline mood was selected as the negative emotion. Average mood scores had a statistically significant effect on the mean ASE scores at the 0.05 level. Higher average negative mood scores of a patient were associated with higher mean ASE scores, whereas higher average positive mood scores were associated with lower mean ASE scores. Model 2: Being at home versus being away from home had a slightly decreasing effect on the mean ASE scores that was not statistically significant at the 0.05 level. Model 3: Being alone versus being with others had an increasing effect on the mean ASE scores that was not statistically significant at the 0.05 level. Model 4: Being alone versus not being alone had a statistically significant increasing effect on the mean ASE scores at the 0.05 level. However, the interaction effect of being alone and being at home indicates that being at home reduces the effect of being alone on the mean ASE scores.

*Marginal R2 and Conditional R2 represent the portions of the variance in the data explained by the fixed effects alone and the fixed and random effects together, respectively.

## Discussion

To the best of our knowledge, this is the first study to explore the relationship between ASEs, general emotions, and social context in the daily lives of adolescents who are at increased risk of developing psychosis. From 27 help-seeking adolescents (12–19 years old), real-time data about the intensity of BSD, mood, and social settings over various time periods (hourly, daily, and weekly over 6 months) were analyzed by linear mixed regression modeling. The results revealed that high levels of ASEs were significantly positively associated with negative emotions and that ASEs were negatively associated with positive emotions. Moreover, being alone was associated with a higher intensity of ASEs than being with others, and being away from home was associated with a higher intensity of ASEs than being at home.

### Anomalous self-experience and its emotional correlates

The strong association between negative mood and intensity of ASE demonstrated in this study is perhaps not surprising. Previous studies have found positive associations between ASE and other specific psychopathological conditions, such as depression and suicidality, which must be considered as correlates of negative emotions. Some of these studies have found strong associations between ASEs and depression and suicidality in individuals with SSD (37), between ASE and anxiety in individuals with panic disorder (38), and between ASE and negative symptoms in CHR samples (14). However, the emotions associated with BSD as potentially distinctive emotional experiences (i.e., the *qualia* that refer to the phenomenology of these emotional experiences) have not been investigated.

Anhedonia (the inability to experience joy or pleasure regardless of the situation) has for some time been regarded as a trait of emotional abnormality in schizophrenia and one of the most prominent negative emotions in this disorder. In fact, psychiatrist Hans Gruhle, nearly 100 years ago, claimed that the essential core of schizophrenia is of an affective nature, a “mood” manifesting itself as a self-disorder, i.e., an unstable and incomplete pre-reflective self-awareness (39). Anhedonia has also been found to be present in first-episode psychosis (FEP) and in CHR samples. Recent studies have demonstrated that the severity of anhedonia in CHR adolescents is not statistically different from that in FEP adolescents and is related to more severe functional impairment and a poorer self-perceived quality of life (40). A study by Yee et al. (41) examined trait emotional characteristics in individuals with schizophrenia and CHR, where participants completed a self-report trait positive and negative affect questionnaire and a clinical symptom interview. The study revealed differences in trait emotions between individuals with schizophrenia and CHR individuals. Whereas the correlates of emotional abnormalities were more integrally linked to primary and idiopathic clinical symptoms in individuals with schizophrenia, these abnormalities were more closely linked to secondary influences such as depression and anxiety in individuals at risk. These studies indicate that there are significant differences in trait emotion across phases of illness and between different patient groups. In another study with the same sample, we examined the temporal stability of ASEs over 6 months (22). The study revealed considerable stability in the level of BSD intensity, indicating that BSD may broadly be considered a trait-like phenomenon even in younger age groups. Taken together, i.e., the strong correlation between ASEs and negative emotions found in the present study and the demonstrated stability of ASEs found in our previous study, these results raise the questions of whether certain emotions may be “trait-specific” for BSD and whether these are more prevalent in CHR adolescents with BSD as compared to CHR adolescents without BSD.

### Anomalous self-experience and social settings

Studies building on the limited work that has used the ESM to examine negative symptoms in CHR youth have demonstrated that negative symptoms differ across different environmental contexts and social relationships (42). Typical ASEs such as derealization, diminished presence, a profound sense of being different from others, and loss of ego boundaries are examples of experiences that may be triggered in social settings. Therefore, we expected that the participants would report a higher intensity of ASEs when being with others than when they were alone. The results showed that being in the company of others compared to being alone was associated with lower levels of ASEs. Previous studies of individuals diagnosed with and at risk of developing psychosis have suggested that social contact is a protective factor, as the severity of different psychotic symptoms increases in the company of strangers and distant others, whereas symptoms are less severe when together with familiar/close others (43, 44). The present study is the first to raise questions about a similar mechanism in adolescents with BSD. While being alone was associated with higher levels of ASEs, this effect seemed to be dependent on the setting/location, as BSD was lower when alone at home vs. alone away from home. We can only speculate whether being away from home may be associated with known stressors such as high expectations at school, pressure to conform with peers, unpredictable situations, and meetings with strangers in public spaces that may increase confusion and anxiety. However, the lower levels of ASEs when being at home may be related to a safe environment and contact with close others (parents and other family members).

### Clinical implications

Despite the increasing research on BSD over the last 20 years, clinical interventions systematically targeting BSD are still lacking. However, although this study was not designed to investigate clinical interventions, our findings suggest that a contextual and dynamic perspective is important in the clinical approach to BSD. The focus on emotions as an agent of change in the interplay between emotion, cognition, and behavior may serve as a tool that is both instructional and exploratory and may increase the patients’ awareness and self-understanding. This may help the patients to downregulate stress and negative emotions, which in turn may lower the intensity of ASEs. Also, the fact that social contact seemed to be related to lower levels of ASE intensity underlines the importance of family work and networking to prevent social isolation and withdrawal, which are present years before the onset of positive symptoms and have been found to predict the risk of having psychotic experiences and of progression to a diagnosable psychotic disorder (45, 46).

## Limitations

This study has several limitations, most importantly the small sample size and the low response rate (34.2%) to the ESM prompts. Missing data are a common and expected challenge for ESM studies, often associated with factors such as long registration periods and debilitating diagnoses under study, such as psychosis; thus, a relatively low response rate was in line with our expectations. We did not analyze factors associated with the unanswered prompts, such as technical problems and skipping prompts in certain settings or times of the day. The registration period of 6 months, which is an extensive period for self-measurement compared to traditional ESM studies, may have been too demanding for our participants and resulted in response fatigue. Furthermore, technical problems with the time algorithm in the app during the 6-month period occurred, which may account for some of the missing answers. As in many ESM studies, response bias cannot be excluded in this study, i.e., less thoughtful or automatic responses over time due to familiarity with the questions, and previous responses may have affected the accuracy of the measurements as the study progressed. Nevertheless, since the 6-month registration period consisted of infrequent measurements at 2-week intervals, this probably made it more challenging to remember previous reports, thus reducing the tendency for automatic responses.

## Data Availability

The raw data supporting the conclusions of this article will be made available by the authors, without undue reservation.
